# Increasing the power of association studies with affected families, unrelated cases and controls

**DOI:** 10.3389/fgene.2013.00200

**Published:** 2013-10-24

**Authors:** William C. L. Stewart, Jane Cerise

**Affiliations:** ^1^Battelle Center for Mathematical Medicine at The Research Institute, Nationwide Children's HospitalColumbus, OH, USA; ^2^Department of Statistics and Pediatrics, Ohio State UniversityColumbus, OH, USA; ^3^Department of Dermatology, Columbia UniversityNY, USA

**Keywords:** POPFAM, sequencing, association, case-control, diabetes

## Abstract

When studying the genetics of inherited diseases, researchers often collect data on affected families, unrelated cases, and healthy controls. However, the joint analysis of such heterogeneous data is difficult, and the simpler analysis of homogeneous subsets is often suboptimal. For example, while case-control tests of association are sensitive to allele frequency differences, the preferential transmission of risk alleles from heterozygous parents to their affected offspring is typically ignored. Similarly, the transmission disequilibrium test (TDT) fails to incorporate the difference in allele frequencies when testing for association. To boost the power of modern genetic studies, we propose POPFAM – a fast and efficient test of association that can accommodate large affected families, unrelated cases, and controls. We use simulations to assess the type I error and power of POPFAM across different genetic models, and minor allele frequencies. For comparison, we examine the power of competing methods: the trend test, a Wald test (equivalent to the TDT), and SCOUT. Our results show that POPFAM maintains the correct type I error, and that it is more powerful than the trend test or the TDT. It performs as well as, or better than the likelihood ratio test SCOUT, which was developed specifically for case-parent/case-control data. Furthermore, when applied to the human leukocyte antigen genotypes of 401 type 1 diabetic families, POPFAM confirmed the previously reported association between DRB1^*^03:01 and microvascular complications (*p* = 0.04). In general, we expect our proposed test to facilitate the identification of clinically important genomic regions, and to better inform the design of follow-up sequencing efforts.

## INTRODUCTION

Many researchers have highly informative, but statistically complicated data. For example, their data may contain a mixture of affected families, independent cases, and controls that are often (but not necessarily) collected at different times. Since these *mixed *data sets contain both population-based and family based lines of evidence, the power to detect an association is potentially increased relative to conventional case-control or transmission disequilibrium tests (TDTs). However, it is difficult to increase power for several reasons. First, the two lines of evidence are usually correlated since they tend to share many (if not all) of the same cases. Second, optimal ways of combining these two lines of evidence are not yet known. In addition, there is always the possibility of spurious associations due to cryptic population stratification (PS). In light of these concerns, many researchers simply report the results of the population-based test and the family based tests separately. This *separate tests *approach has three main drawbacks. First, the component tests are often less powerful than tests that combine the two sources of information. Second, it is difficult to summarize the overall evidence for association when one component test is significant and the other is not. Third, if the case-control test is significant, then a significant family based test will often be interpreted as assurance that the case-control finding was not spurious (i.e., the result of cryptic PS). However, since modern case-control studies can substantially reduce the negative effect of cryptic PS (Price et al., 2006; Epstein et al., 2007; Li et al., 2010; Thornton and McPeek, 2010), the confirmatory role of the family based test (and hence the need for separate tests) has lost much of its original appeal.

In contrast to the *separate tests *approach, others advocate a *combined test *approach. Broadly speaking, a combined test can be classified as either likelihood-based, or meta-analysis. The meta-analysis methods combine summary statistics derived from possibly overlapping, and often exhaustive subsets of the mixed data. Some of the earliest mixed data methods (Clayton, 1999; Whittemore and Tu, 2000; Epstein et al., 2005; Putter et al., 2005) used the likelihoods of genotype relative risk (GRR) parameters to test for association. Many of these methods were direct extensions of the Schaid and Sommer (1993) CPG-likelihood, which was designed for case-parent genotype data (i.e., trios). Shortly thereafter, other authors began to use the meta-analysis framework (Putter et al., 2005; Bagos and Nikolopoulos, 2007; Chen and Lin, 2008; Mirea et al., 2012), which focuses on combinations (esp. linear combinations) of summary statistics. In addition to being almost as powerful as the likelihood-based methods that pre-date them, the meta-analysis methods offer several other attractive features as well. First, the primary genotype data are no longer needed. Second, handling more elaborate designs, such as extended families and measured covariates is easier. Third, parametric and non-parametric procedures are easily incorporated. Nevertheless, a disadvantage of almost all *existing *mixed data methods is that they require specification *a priori *of a genetic model (e.g., additive, dominant, multiplicative, or recessive). One notable exception is the non-parametric method of Bagos and Nikolopoulos (2007). However, for this method, the summary statistics are so restrictive that extensions to large families and/or additional controls with measured covariates are difficult at best. For a more detailed review of association methods that analyze the genotypes of case-parent and unrelated individuals, see Infante-Rivard et al. (2009).

Using the theory of linear models, Chen and Lin (2008) proposed a combined test that is the linear combination of a family based test and a population-based test of association. Furthermore, if the component tests (i.e., the family based test and the population-based test) are symmetrically distributed about the same mean under the alternative, then their test is indeed *optimal *(i.e., most powerful). However, this condition is rarely met, and in practice the optimal weights will depend on the marginal power of each component test. Although the marginal power of any test is typically not known *a priori*, the trend test (Armitage, 1955) and the TDT (Spielman et al., 1993) have been shown to have similar power across a wide range of genetic models (McGinnis et al., 2002). Therefore, when the component tests are equivalent to the trend test and to the TDT, equal weights may be used. However, when one component test has substantially higher power than the other, a weight that varies inversely in proportion to an appropriate measure of sample size tends to work well in practice (see Methods).

Recall that for most mixed data sets, the population-based and family based tests will use the genotypes of a shared set of cases, which means that the two tests will be correlated. Like Chen and Lin (2008), our proposed test: POPFAM, is also a linear combination of two component tests, and we use a scaling factor that depends on the correlation to ensure that our test is normally distributed [i.e., *N*(0,1)] under the null hypothesis of no association. However, unlike Chen and Lin (2008), and unlike SCOUT (Epstein et al., 2005) – a competing likelihood ratio test, POPFAM does not make any assumptions about the underlying genetic model. Furthermore, POPFAM is flexible, in that large families (with or without missing data), publicly available controls, and measured covariates are easily incorporated. Moreover, its full potential is perhaps best realized when one incorporates multipoint linkage statistics like EAGLET (Stewart et al., 2010, 2011), KELVIN (Vieland et al., 2011), and/or MORGAN (Thompson, 2000). For example, a multipoint linkage method could provide weights for POPFAM *p-values* in a weighted false discovery rate (FDR) approach. Similarly, by restricting the association analysis to regions beneath linkage peaks, these same multipoint linkage methods could also reduce the multiple testing burden.

## METHODS

For ease of exposition, let’s suppose that we have genotype data at a single nucleotide polymorphism (SNP) on affected trios and controls sampled from a genetically homogenous population (i.e., there is no PS), and let’s assume that every case is an affected offspring with unphenotyped parents. Later, we will discuss how related cases can be incorporated into the analysis. Define a population-based test of association *P *that depends on the genotypes of cases and controls, and a family based test *F *that depends on the genotypes of the same cases and their relatives. Our proposed test, POPFAM, is

$$[wP+(1-w)F]/\sqrt{w^{2}\cdot Var(P)+{\left(1-w\right)}^{2}\cdot Var(F)+2w(1-w)\cdot \tau },$$

where τ denotes Cov(*P*,*F*). The optimal weight *w* depends on the relative power of *P *and *F*. However, since this information is rarely known *a priori*, it is often useful to define *w* on the basis of sample size (e.g., *w *≡ *A*/(*A* + *H*) with *A *defined as one half of the harmonic mean of cases and controls, and *H* denoting the number of heterozygous parents). When the mixed data are (or can be treated as) case-parent trios and controls (which is often the case), we have a closed-form algebraic expression for τ (see **Appendix A**) that permits the fast and accurate computation of *p-values *across the genome.

POPFAM uses the trend (i.e., case-control) test for *P, *and the Wald test of Mendelian segregation for* F*. Formally, for *M* controls, *N* case-parent trios with *X* ≤ *N* trios having at least one heterozygous parent, let *T*_i_ count the number of “1” alleles transmitted from a heterozygous parent to his/her affected offspring, and let *H* ≤ 2*N *be the total number of heterozygous parents across all *N *trios. Further, let *R*_j_ and *S*_k_ count the number of “1” alleles in the *j*th case and the *k*th control, for *j* = 1, ⋯, *N*, and *k* = 1, ⋯, *M*. Then, the trend test of proportions (denoted by *P*) and the Wald test (denoted by *F*) are

(1) $$P=\frac{\left(\Sigma R_{j}/N-\Sigma S_{k}/M\right)}{\sqrt{\sigma^{2}\left(N^{-1}+M^{-1}\right)}}\,$$

(2) $$\;F=\sqrt{4H}(\Sigma T_{i}/H-0.5),$$

where *σ*^2^ = 2*p*(1 - *p*) assuming Hardy-Weinberg equilibrium (HWE). If HWE does not hold, then σ ^2^ can be estimated with the method of moments by [4g_2_ + g_1_ - (N + M)$\overset{¯}{g}$^2^ ] . / (N + M) . Here, *g*_1_ and *g*_2_ count the number of cases and controls with 1 and 2 alleles of type “1,” and $\overset{¯}{g}$=(2g_2_ +g_1_ )/(N+M). The minor allele frequency (MAF) is denoted by *p. *Note that the trend test is equivalent to the standard two-sample test of proportions when HWE holds. With this general framework, missing data, large families, and measured covariates can, in principle, be handled by considering other choices of *P *and *F *such as logistic regression, GDT (Chen et al., 2009) and/or PDT (Martin et al., 2000). Furthermore, the limiting distribution of POPFAM under the null hypothesis of no association is *N*(0,1) since *P *and* F* are asymptotically normal with mean zero under the null.

Furthermore, to safeguard against the negative effects of cryptic PS, users could for example base the component test *P *on the scaled slope coefficient from an EIGENSTRAT (Price et al., 2006) analysis. In doing so, the top principal components (which are estimated from the observed genotypes) mitigate the negative effects (if any) of cryptic PS. Alternatively one could opt to use the test statistic implemented in ROADTRIPS (Thornton and McPeek, 2010) instead. In addition to correcting for cryptic PS, this statistic also corrects for any unreported relatedness such as second or third cousin relationships. However, the limiting distribution of the test implementedin ROADTRIPS is chi squared, which means that the resulting POPFAM *p-values* would have to be estimated empirically.

## DATA: SIMULATED AND REAL

To assess the power (and type I error) of POPFAM, and to examine the sensitivity of a competing parametric method named SCOUT (Epstein et al., 2005), we simulated SNP genotypes for 200 case-parent trios and 200 controls under the null hypothesis of no association, and under six different alternative hypotheses (i.e., scenarios) that were neither additive, dominant, multiplicative, nor recessive (**Table 1**). We chose to compare POPFAM to SCOUT, since the latter has comparable performance to several existing mixed data methods (Epstein et al., 2005; Guo et al., 2009). For the six scenarios, the correlation was fixed at D′ = 0.80, the disease prevalence was set to 5 percent with a 4.5 percent phenocopy rate (i.e., wildtype homozygotes were affected with probability 0.045). We varied the disease allele frequency (DAF) from 0.03 to 0.30, although we were particularly interested in SNPs with MAFs between 0.03 and 0.12, since many variants in this range have escaped detection by existing (i.e., less powerful) association methods, and since many of these variants are expected to have clinically meaningful effects (Dickson et al., 2010). Type I error was fixed at 5%, and power was estimated from 1000 replicates. The MAF of the SNP was always equal to the DAF for each scenario. Note that since our simulations use an equal number of cases and controls (i.e., *N *= *M*), and since we chose the trend test and the Wald test of Mendelian segregation for *P *and* F* respectively, the two tests have comparable power (McGinnis et al., 2002). As such, the POPFAM-related weight was set to ½. Note that, when sample size was used to compute the weight (see Methods), it ranged from 0.75 to 0.5 as the MAF increased from 0.03 to 0.30. The results were qualitatively the same whether the weight was fixed or allowed to vary (data not shown).

**Table 1:** Genetic models for simulated data.

Scenario	DAF	Pr(Aff\textbar D/d)	Pr(Aff\textbar D/D)
S1	0.03	0.125	0.440
S2	0.06	0.084	0.218
S3	0.09	0.066	0.237
S4	0.12	0.060	0.173
S5	0.20	0.053	0.100
S6	0.30	0.050	0.080

*Here, “d” denotes the wild type allele at the disease locus, and “D” denotes the causal allele thatincreases risk. DAF is the disease allele frequency.
*

A recent case-control study among type 1 diabetics (Lipner et al., 2013), reported a protective effect of DRB1*03:01 for microvascular complications (e.g., retinopathy, nephropathy, and/or neuropathy) (*p* < 0.045). Therefore, to demonstrate the utility of POPFAM, we analyzed 117 case-parent trios, and 277 controls at the DRB1*03 marker of the human leukocyte antigen (HLA) class II locus. In our analysis, two lines of correlated information were combined (1) the DRB1*03:01 allele frequency difference between type 1 diabetics with and without complications, and (2) the preferential transmission of DRB1*03:01 alleles from heterozygous parents to type 1 diabetic offspring without complications. Note that, in this example, the second line of evidence is no longer straightforward in that the excess transmission measured by *F* is no longer measured relative to one half. Instead, it must be measured relative to the over-transmission of DRB1*03:01 alleles to type 1 diabetics with complications. SCOUT cannot accommodate such an analysis, and this line of evidence was ignored in the original study that first reported the association between DRB1^*^03 and microvascular complications (e.g., retinopathy, nephropathy, and/or neuropathy).

## RESULTS

From **Table 2**, we can see that all three tests (our proposed test POPFAM, the population-based test *P*, and the family based test *F*) are valid in that their type I errors are controlled. Moreover, for the six alternative hypotheses considered (i.e., scenarios 1–6), POPFAM outperforms *P *and *F*, and usually outperforms SCOUT in terms of power (**Table 3**). Note that, since the program SCOUT requires specification *a priori *of a genetic model (e.g., additive, dominant, recessive, or multiplicative), it is difficult to compare SCOUT to a non-parametric test like POPFAM. Nevertheless, POPFAM outperforms SCOUT in 5 of 6 scenarios even if the best fitting genetic model were known. Also, if one happens to choose the worst fitting genetic model, then even the component tests: *P *and *F* outperform SCOUT.

**Table 2:** Type I error of POPFAM, *P* and *F.*

Test	5%	1%
POPFAM	**0.045**	**0.009**
*P*	0.054	0.009
*F*	0.064	0.007

*Type I errors are based on 1000 null replicates. POPFAM is our proposed test, *P* is the trend (i.e., case-control) test, and *F *is the Wald equivalent of the TDT.
*

In our secondary data analysis of type 1 diabetics, POPFAM confirmed the association between DRB1^*^03 and microvascular complications (*p* = 0.04). Specifically, the *p-value* of the case-control test was 0.045, whereas the *p-value* for POPFAM (i.e., the test that integrates population-based and family-based lines of evidence) was 0.04. Note that, the family-based test alone was not significant (*p* < 0.12), but when combined with the case-control test the overall evidence for association increased. This same phenomenon also occurs in the simulated data, and it explains why POPFAM is always more powerful than *P* or* F* alone **Table 3**.

**Table 3:** Power (%) to detect an association.

Scenario	POPFAM	*P*	*F*	SCOUT^*^
S1	**91.0**	88.0	74.8	[29.1, 84.1]
S2	**72.6**	63.3	54.5	[24.1, 68.1]
S3	**69.0**	60.8	55.0	[49.0, 66.5]
S4	**57.0**	47.1	43.0	[38.3, 54.0]
S5	**41.7**	31.2	32.4	[25.2, 41.0]
S6	**30.3**	24.8	23.9	[15.9, 33.2]

* For each scenario, the power of POPFAM is given in bold, while the minimum and maximum power of SCOUT across additive, dominant, multiplicative, and recessive models is shown in brackets. The scenarios (S1–S6) are defined in **Table 1**. The power for component tests, *P *(population-based) and *F *(family based) are also shown for comparison.

## DISCUSSION

We have demonstrated that POPFAM–our fast, flexible, and nonparametric test of association increases power and controls the type I error. Relative to test that rely either upon population-based or family-based data alone, POPFAM can substantially boost power with gains as large as 14%. We are particularly interested in the increased power for SNPs with MAFs between 0.03 and 0.12 because the power to detect associations using SNPs in this class is quite low (Pelak et al., 2010), and because some SNPs in this class are undoubtedly highly correlated with clinically meaningful disease-related variants. In addition, we have shown that POPFAM performs as well as, or better than SCOUT. This is important, because SCOUT is generally better than CPG (Schaid and Sommer, 1993) and Chen and Lin (2008), with only a slight decline in power relative to Guo et al. (2009).

The meta-analysis literature in statistical genetics and epidemiology has been somewhat misleading with respect to claims of optimally weighted linear combinations of component tests. The problem arises from the fact that several authors have used the term *optimal* in the context of parameter estimation (Bagos and Nikolopoulos, 2007; Chen and Lin, 2008), whereas readers tend to interpret the word in the context of hypothesis testing. In the context of hypothesis testing, the word *optimal *is generally taken to mean *most powerful* (as opposed to *most accurate*). In the special case that both summary statistics are normally distributed about the same mean under the alternative hypothesis of association, there is no confusion because both interpretations yield the same weights. However, this condition is rarely met, and in practice the optimal weight will tend to depend on unknown parameters under the alternative (i.e., the marginal power of each summary statistic).

POPFAM is more than a test of association; it is a testing framework. The attractive features that POPFAM brings to the case-parent/case-control design are likely to transfer to more complex designs as well (i.e., designs that involve covariates, and that may require a larger and more diverse set of component tests). For example, we have already successfully applied POPFAM to a real mixed data set with large, extended families and publicly available controls (i.e., HapMap CEU samples; data not shown). In principle, these wider applications can be carried out in one of two ways: (1) decompose the large families into trios whenever both parents provide genotype data, or (2) choose a family based test statistic that can accommodate large families (e.g., GDT). To facilitate power analyses, POPFAM can also simulate case-parent/case-control genotype data conditional on the affectedness status of each case and control. Overall, POPFAM represents the next logical step for detecting genetic associations with disease from the analysis (or re-analysis) of mixed data. The software is freely available from the web at:  and is distributed as part of the EAGLET suite.

## Conflict of Interest Statement

The authors declare that the research was conducted in the absence of any commercial or financial relationships that could be construed as a potential conflict of interest.

## APPENDIX

### COVARIANCE OF *P* AND *F* UNDER THE NULL

For ease of exposition, let *p, σ*^2^*, H, M, N, T*_i_, *R*_j_, *S*_k_, *P*, *F *and *X *be defined as before (see Methods) and define $\theta \equiv \sqrt{\sigma^{2}\left(N^{-1}+M^{-1}\right)}$. Without loss of generality, we shall assume that the “test allele” is also the minor allele. Therefore, under the null hypothesis of no association, *Cov*(*P,F*) is:

$$=Cov\left[\left(\Sigma R_{j}/N-\Sigma S_{k}/M\right)/\theta ,\sqrt{4H}\left(\Sigma T_{i}/H-½\right)\right]$$

$$=E\left[\left(\Sigma R_{j}/N-\Sigma S_{k}/M\right)/\theta .\sqrt{4H}\left(\Sigma T_{i}/H-½\right)\right]\,\left(EP=EF=0\right)$$

$$={\left(N\theta \right)}^{-1}\cdot E[(\Sigma R_{j}\cdot \sqrt{4H})\left(\Sigma T_{i}/H-½\right)]\left(\Sigma S_{k}⊥F\right)$$

$$={\left(N\theta \right)}^{-1}\cdot E\left[E\left(\Sigma R_{j}\cdot \sqrt{4H}\left(\Sigma T_{i}/H-½\right)|H,X\right)\right]$$

Now, let *v *index the informative trios (i.e., trios for which there is at least one heterozygous parent). To compute the iterated expectation, it is also useful to define *R*_v_ = *R*_j_ whenever the *j*th case is a member of the *v*th informative trio. Similarly, we also need to keep track of *Q*_v_ = the number of transmitted “1” alleles, and *D*_v_ = the number of heterozygous parents, where both *Q*_v_ and *D*_v_ are defined relative to the *v*th informative trio. Therefore, $\Sigma T_{i}=\Sigma Q_{v},H=\Sigma D_{v},$ and $(\Sigma R-\Sigma Q_{v})⊥F.$ Continuing with the computation of *Cov*(*P,F*), we now have that the last expression

$$={\left(N\theta \right)}^{-1}\cdot E[\frac{2}{\sqrt{H}}E\left(\Sigma R_{v}\cdot \Sigma \left(Q_{v}-D_{v}/2\right)|H,X\right)]$$

$$={\left(N\theta \right)}^{-1}\cdot E\left[\frac{2}{\sqrt{H}}E\left(R_{v}\cdot \left(Q_{v}-D_{v}/2\right)\right)\right](H,X)⊥\left(Q_{v}-D_{v}/2\right)$$

Now, the inner expectation is easily computed using the unconditional probabilities of the seven possible genotypic configurations for an informative trio. The outer expectation is tedious, but tractable since *X ~ Bin*(*N*, *p*′)/Pr(*X *> 0 ), and since *H - x* ∣ *X* ß *Bin*(*x*, *p*″), where *p*′ is the unconditional probability of a randomly chosen trio being informative, and *p*″ is the conditional probability of a double heterozygote mating type given that the trio is informative.
